# Tetra­kis(μ-4-methoxy­benzoato)bis­[(4-methoxy­benzoato)(1,10-phenan­throline)terbium(III)]

**DOI:** 10.1107/S1600536809037751

**Published:** 2009-09-26

**Authors:** Zhi-Hua Gao, Hao Wang, Jing-Yu He, Rui-Fen Wang

**Affiliations:** a College of Materials Science & Engineering, Beijing University of Technology, Beijing 100124, People’s Republic of China; b College of Chemistry, Hengshui University, Hengshui 053000, People’s Republic of China; c College of Life Science & Bio-engineering, Beijing University of Technology, Beijing 100124, People’s Republic of China; d College of Chemistry & Materials Science, Hebei Normal University, Shijiazhuang 050016, People’s Republic of China

## Abstract

In the title dinuclear complex, [Tb_2_(C_8_H_7_O_3_)_6_(C_12_H_8_N_2_)_2_], each Tb^III^ ion is eight-coordinated by two N atoms from a 1,10-phenanthroline ligand and six O atoms from the carboxyl­ate groups of five 4-methoxy­benzoate ligands in a distorted square-anti­prismatic geometry. All six 4-methoxy­benzoate ligands act in a bidentate mode, two coordinating to one Tb center each and the other four bridging two Tb centers [Tb⋯Tb separation = 4.3144 (6) Å]. In the crystal, inter­molecular π–π inter­actions between the aromatic rings of 1,10-phenanthroline and 4-methoxy­benzoate ligands [centroid–centroid distance = 3.742 (9) Å] link two mol­ecules into a centrosymmetric dimer. Weak inter­molecular C—H⋯O hydrogen bonds help to consolidate the crystal packing.

## Related literature

For general background to lanthanide complexes, see: Liu *et al.* (2004 ▶); Guo *et al.* (2005 ▶); Zhang *et al.* (2005 ▶). For a related structure, see: Wang *et al.* (2006 ▶).
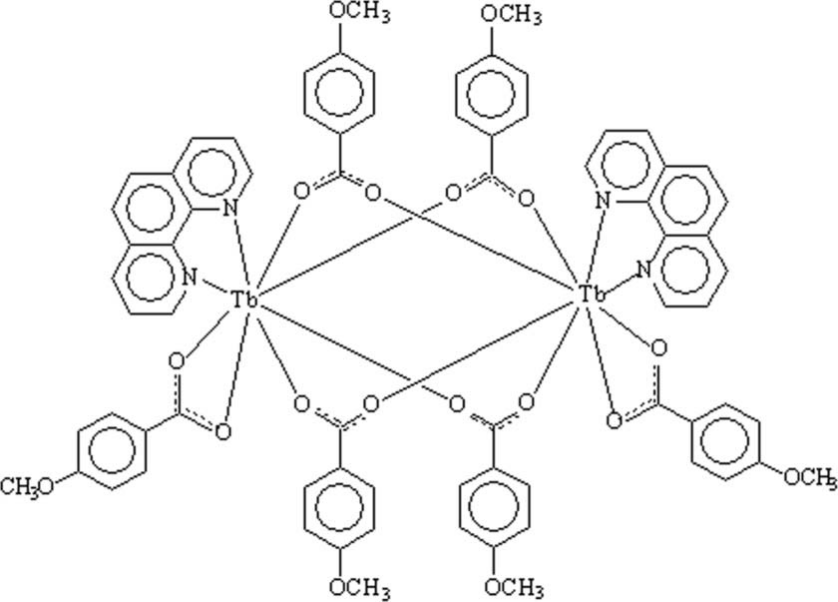


## Experimental

### 

#### Crystal data

[Tb_2_(C_8_H_7_O_3_)_6_(C_12_H_8_N_2_)_2_]
*M* *_r_* = 1585.06
Monoclinic, 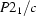

*a* = 18.055 (2) Å
*b* = 15.1404 (12) Å
*c* = 25.954 (3) Å
β = 109.070 (2)°
*V* = 6705.3 (11) Å^3^
*Z* = 4
Mo *K*α radiation
μ = 2.17 mm^−1^
*T* = 298 K
0.28 × 0.26 × 0.25 mm

#### Data collection

Bruker APEXII CCD area-detector diffractometer
Absorption correction: multi-scan (*SADABS*; Sheldrick, 1996 ▶) *T*_min_ = 0.582, *T*_max_ = 0.613
33244 measured reflections
11810 independent reflections
8197 reflections with *I* > 2σ(*I*)
*R*_int_ = 0.048

#### Refinement

*R*[*F* ^2^ > 2σ(*F* ^2^)] = 0.043
*wR*(*F* ^2^) = 0.112
*S* = 1.11
11810 reflections
865 parameters
H-atom parameters constrained
Δρ_max_ = 1.52 e Å^−3^
Δρ_min_ = −1.11 e Å^−3^

### 

Data collection: *SMART* (Bruker, 1998 ▶); cell refinement: *SAINT* (Bruker, 1998 ▶); data reduction: *SAINT*; program(s) used to solve structure: *SHELXS97* (Sheldrick, 2008 ▶); program(s) used to refine structure: *SHELXL97* (Sheldrick, 2008 ▶); molecular graphics: *SHELXTL* (Sheldrick, 2008 ▶); software used to prepare material for publication: *SHELXTL*.

## Supplementary Material

Crystal structure: contains datablocks I, global. DOI: 10.1107/S1600536809037751/cv2614sup1.cif

Structure factors: contains datablocks I. DOI: 10.1107/S1600536809037751/cv2614Isup2.hkl

Additional supplementary materials:  crystallographic information; 3D view; checkCIF report

## Figures and Tables

**Table 1:** Hydrogen-bond geometry (Å, °)

*D*—H⋯*A*	*D*—H	H⋯*A*	*D*⋯*A*	*D*—H⋯*A*
C32—H32*B*⋯O6^i^	0.96	2.51	3.059 (13)	116
C51—H51⋯O9^ii^	0.93	2.41	3.317 (10)	164
C56—H56⋯O15^iii^	0.93	2.56	3.341 (9)	143
C60—H60⋯O15^iii^	0.93	2.48	3.280 (10)	144
C69—H69⋯O16^iv^	0.93	2.52	3.285 (9)	139

Symmetry codes: (i) 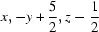; (ii) 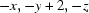; (iii) 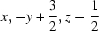; (iv) 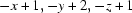.

**Fig. 1.**
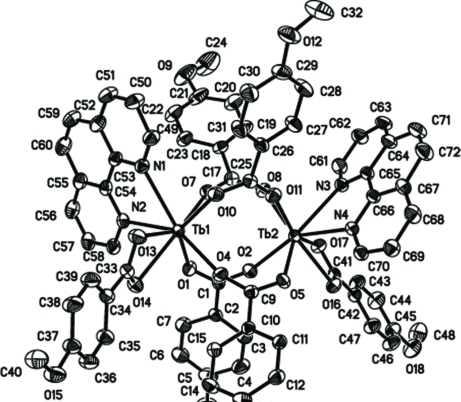
The structure of the title complex, showing the atom-numbering scheme. Displacement ellipsoids are drawn at the 30% probability level and H atoms have been omitted for clarity.

## supplementary crystallographic information

### Comment

Lanthanide complexes of organic ligands, especially Eu^III^ and Tb^III^ complexes, often show excellent luminescence (Guo *et al.*, 2005; Liu *et al.*, 2004; Zhang *et al.*, 2005). The choice of appropriate ligands is a key step in determining the luminescent characteristics of the complexes. Aromatic carboxylic acids are strong ligands to coordinate lanthanide metals. In this work, we present the title compound (I) - an efficient luminescent terbium complex with 4-methoxybenzoato and 1,10-phenanthroline ligands.

In each molecule of (I) (Fig. 1), two Tb^III^ ions are linked by four carboxylate groups in a bidentate bridging mode. The intramolecular distance between the two Tb^III^ ions is 4.3144 (6) Å. Each Tb^III^ ion resides in an eight-coordinate distorted square antiprismatic environment, with four of the coordination sites occupied by the O atoms of the bridging carboxylates, two by the O atoms of the terminal bidentate carboxylates, and the remaining positions occupied by two N atoms of a 1,10-phenanthroline molecule. The Tb—O distances are in the range of 2.255 (5)–2.469 (5) Å, of which the Tb—O bonds formed by the terminal bidentate carboxylate groups [average distance = 2.427 (3) Å] are longer than those formed by the bridging carboxylates [average distance = 2.323 (5) Å]. The Tb—N distances are in the range of 2.575 (6)–2.624 (6) Å. The five-membered chelate rings containing two N atoms and the terbium ion are nearly coplanar with the 1,10-phenanthroline molecule [dihedral angle = 4.9 (2) and 2.3 (5)°].

In the crystal, intermolecular π-π interactions between the aromatic rings of 1,10-phenanthroline and 4-methoxybenzoato ligands [centroid-to-centroid distance of 3.742 (9) Å] link two molecules into centrosymmetric dimer. Weak intermolecular C—H···O hydrogen bonds (Table 1) help to consolidate the crystal packing.

### Experimental

The title complex was prepared *via* a modification of previously reported method (Wang *et al.*, 2006). Stoichiometric amounts of terbium chloride, 4-methoxybenzoic acid and 1,10-phenanthroline were dissolved separately in 95% ethanol. The pH value of the 4-methoxybenzoic acid was adjusted to the range of 6–7 with NaOH solution. The solutions of the two ligands were mixed and the mixture was added dropwise to the ethanolic TbCl_3_ solution; a white precipitate then formed. The mixture was stirred for 4 h, and then was filtered. The filtrate was slowly evaporated at room temperature, and colorless crystals suitable for X-rayinvestigation were obtained. Analysis calculated for C_72_H_58_N_4_O_18_Tb_2_: C 54.50, H 3.66, N 3.53%; found: C 54.37, H 3.77, N 3.43%.

### Refinement

H atoms were placed in geometrically calculated positions and allowed to ride on their parent atoms, with C—H = 0.93 (CH) and 0.96 (CH_3_)Å and with *U*iso(H) = 1.2(or 1.5 for methyl)*U*eq(C).

### Crystal data

**Table:**

[Tb_2_(C_8_H_7_O_3_)_6_(C_12_H_8_N_2_)_2_]	*F*(000) = 3168
*M**_r_* = 1585.06	*D*_x_ = 1.570 Mg m^−^^3^
Monoclinic, *P*2_1_/*c*	Melting point: 513 K
Hall symbol: -P 2ybc	Mo *K*α radiation, λ = 0.71073 Å
*a* = 18.055 (2) Å	Cell parameters from 9702 reflections
*b* = 15.1404 (12) Å	θ = 2.3–25.7°
*c* = 25.954 (3) Å	µ = 2.17 mm^−^^1^
β = 109.070 (2)°	*T* = 298 K
*V* = 6705.3 (11) Å^3^	Block, colourless
*Z* = 4	0.28 × 0.26 × 0.25 mm

### Data collection

**Table:**

Bruker APEXII CCD area-detector diffractometer	11810 independent reflections
Radiation source: fine-focus sealed tube	8197 reflections with *I* > 2σ(*I*)
graphite	*R*_int_ = 0.048
φ and ω scans	θ_max_ = 25.0°, θ_min_ = 1.2°
Absorption correction: multi-scan (*SADABS*; Sheldrick, 1996)	*h* = −21→16
*T*_min_ = 0.582, *T*_max_ = 0.613	*k* = −18→17
33244 measured reflections	*l* = −30→30

### Refinement

**Table:**

Refinement on *F*^2^	Primary atom site location: structure-invariant direct methods
Least-squares matrix: full	Secondary atom site location: difference Fourier map
*R*[*F*^2^ > 2σ(*F*^2^)] = 0.043	Hydrogen site location: inferred from neighbouring sites
*wR*(*F*^2^) = 0.112	H-atom parameters constrained
*S* = 1.11	*w* = 1/[σ^2^(*F*_o_^2^) + (0.0296*P*)^2^ + 25.2631*P*] where *P* = (*F*_o_^2^ + 2*F*_c_^2^)/3
11810 reflections	(Δ/σ)_max_ = 0.001
865 parameters	Δρ_max_ = 1.52 e Å^−^^3^
0 restraints	Δρ_min_ = −1.11 e Å^−^^3^

### Special details

**Table:**

Geometry. All e.s.d.'s (except the e.s.d. in the dihedral angle between two l.s. planes) are estimated using the full covariance matrix. The cell e.s.d.'s are taken into account individually in the estimation of e.s.d.'s in distances, angles and torsion angles; correlations between e.s.d.'s in cell parameters are only used when they are defined by crystal symmetry. An approximate (isotropic) treatment of cell e.s.d.'s is used for estimating e.s.d.'s involving l.s. planes.
Refinement. Refinement of *F*^2^ against ALL reflections. The weighted *R*-factor *wR* and goodness of fit *S* are based on *F*^2^, conventional *R*-factors *R* are based on *F*, with *F* set to zero for negative *F*^2^. The threshold expression of *F*^2^ > σ(*F*^2^) is used only for calculating *R*-factors(gt) *etc*. and is not relevant to the choice of reflections for refinement. *R*-factors based on *F*^2^ are statistically about twice as large as those based on *F*, and *R*- factors based on ALL data will be even larger.

### Fractional atomic coordinates and isotropic or equivalent isotropic displacement parameters (Å^2^)

**Table:**

	*x*	*y*	*z*	*U*_iso_*/*U*_eq_	
Tb1	0.156242 (19)	0.90029 (2)	0.302963 (13)	0.03315 (10)	
Tb2	0.403401 (19)	0.95496 (2)	0.344664 (13)	0.03370 (10)	
N1	0.0528 (3)	0.9701 (4)	0.2167 (2)	0.0458 (16)	
N2	0.0703 (3)	0.7931 (4)	0.2311 (2)	0.0447 (15)	
N3	0.4795 (3)	1.0566 (4)	0.2998 (2)	0.0444 (15)	
N4	0.4703 (3)	1.0944 (4)	0.3993 (2)	0.0416 (14)	
O1	0.2305 (3)	0.7688 (3)	0.3130 (2)	0.0477 (13)	
O2	0.3542 (3)	0.8143 (3)	0.3423 (2)	0.0483 (13)	
O3	0.3982 (4)	0.4209 (4)	0.4281 (3)	0.088 (2)	
O4	0.2428 (3)	0.9104 (3)	0.3880 (2)	0.0485 (13)	
O5	0.3666 (3)	0.9543 (3)	0.42385 (18)	0.0434 (12)	
O6	0.2860 (3)	0.8758 (4)	0.6389 (2)	0.0623 (16)	
O7	0.2204 (3)	0.9192 (3)	0.24124 (19)	0.0477 (13)	
O8	0.3457 (3)	0.9371 (3)	0.24865 (19)	0.0486 (13)	
O9	0.1865 (4)	0.9014 (5)	−0.0066 (2)	0.096 (2)	
O10	0.1826 (3)	1.0530 (3)	0.31167 (19)	0.0469 (13)	
O11	0.3095 (3)	1.0625 (3)	0.3206 (2)	0.0486 (13)	
O12	0.1910 (4)	1.4464 (4)	0.2350 (3)	0.0800 (19)	
O13	0.0497 (3)	0.9445 (4)	0.3315 (2)	0.0551 (14)	
O14	0.0910 (3)	0.8093 (4)	0.3544 (2)	0.0504 (13)	
O15	−0.0952 (3)	0.8809 (4)	0.5200 (2)	0.0610 (16)	
O16	0.5180 (3)	0.8924 (3)	0.41040 (19)	0.0475 (13)	
O17	0.5050 (3)	0.8663 (3)	0.3247 (2)	0.0492 (13)	
O18	0.8270 (3)	0.6680 (4)	0.4474 (3)	0.0770 (19)	
C1	0.3028 (5)	0.7558 (5)	0.3359 (3)	0.0441 (18)	
C2	0.3292 (4)	0.6660 (5)	0.3570 (3)	0.0450 (19)	
C3	0.4031 (5)	0.6515 (5)	0.3938 (3)	0.056 (2)	
H3	0.4385	0.6981	0.4037	0.067*	
C4	0.4249 (6)	0.5694 (6)	0.4161 (4)	0.067 (3)	
H4	0.4747	0.5607	0.4411	0.080*	
C5	0.3724 (6)	0.4994 (6)	0.4013 (4)	0.066 (2)	
C6	0.3015 (6)	0.5116 (6)	0.3641 (4)	0.067 (2)	
H6	0.2673	0.4641	0.3528	0.080*	
C7	0.2791 (5)	0.5946 (5)	0.3425 (3)	0.059 (2)	
H7	0.2292	0.6026	0.3176	0.071*	
C8	0.3430 (7)	0.3499 (7)	0.4175 (5)	0.108 (4)	
H8A	0.2956	0.3700	0.4228	0.163*	
H8B	0.3647	0.3020	0.4421	0.163*	
H8C	0.3318	0.3301	0.3806	0.163*	
C9	0.3033 (5)	0.9272 (5)	0.4283 (3)	0.0411 (18)	
C10	0.2972 (4)	0.9135 (5)	0.4836 (3)	0.0389 (17)	
C11	0.3432 (4)	0.9604 (5)	0.5284 (3)	0.0436 (18)	
H11	0.3795	1.0009	0.5243	0.052*	
C12	0.3360 (5)	0.9479 (5)	0.5788 (3)	0.0485 (19)	
H12	0.3650	0.9823	0.6082	0.058*	
C13	0.2861 (5)	0.8848 (5)	0.5860 (3)	0.048 (2)	
C14	0.2393 (5)	0.8365 (5)	0.5424 (3)	0.051 (2)	
H14	0.2048	0.7943	0.5472	0.061*	
C15	0.2451 (4)	0.8523 (5)	0.4910 (3)	0.0480 (19)	
H15	0.2132	0.8211	0.4611	0.058*	
C16	0.2457 (6)	0.8031 (7)	0.6507 (4)	0.083 (3)	
H16A	0.1903	0.8115	0.6339	0.124*	
H16B	0.2578	0.7981	0.6895	0.124*	
H16C	0.2614	0.7501	0.6368	0.124*	
C17	0.2743 (5)	0.9302 (5)	0.2215 (3)	0.0467 (19)	
C18	0.2514 (5)	0.9320 (6)	0.1608 (3)	0.049 (2)	
C19	0.3025 (5)	0.9575 (6)	0.1344 (3)	0.062 (2)	
H19	0.3517	0.9789	0.1547	0.075*	
C20	0.2823 (5)	0.9521 (7)	0.0780 (4)	0.073 (3)	
H20	0.3168	0.9712	0.0604	0.088*	
C21	0.2100 (6)	0.9176 (7)	0.0487 (4)	0.076 (3)	
C22	0.1562 (5)	0.8970 (7)	0.0733 (3)	0.068 (3)	
H22	0.1060	0.8787	0.0528	0.081*	
C23	0.1776 (5)	0.9039 (6)	0.1288 (3)	0.058 (2)	
H23	0.1413	0.8890	0.1458	0.069*	
C24	0.2438 (7)	0.9014 (10)	−0.0327 (4)	0.122 (5)	
H24A	0.2626	0.9605	−0.0337	0.184*	
H24B	0.2212	0.8796	−0.0693	0.184*	
H24C	0.2867	0.8640	−0.0130	0.184*	
C25	0.2427 (5)	1.0959 (5)	0.3102 (3)	0.0431 (18)	
C26	0.2319 (4)	1.1894 (5)	0.2925 (3)	0.0452 (19)	
C27	0.2883 (5)	1.2327 (5)	0.2770 (3)	0.055 (2)	
H27	0.3350	1.2039	0.2797	0.066*	
C28	0.2765 (5)	1.3190 (6)	0.2573 (4)	0.063 (2)	
H28	0.3149	1.3472	0.2466	0.076*	
C29	0.2087 (6)	1.3622 (6)	0.2539 (4)	0.063 (2)	
C30	0.1529 (5)	1.3209 (6)	0.2701 (4)	0.063 (2)	
H30	0.1070	1.3506	0.2680	0.075*	
C31	0.1639 (5)	1.2363 (5)	0.2892 (3)	0.054 (2)	
H31	0.1254	1.2094	0.3004	0.065*	
C32	0.2422 (7)	1.4885 (7)	0.2114 (5)	0.101 (4)	
H32A	0.2933	1.4936	0.2382	0.152*	
H32B	0.2224	1.5462	0.1989	0.152*	
H32C	0.2455	1.4541	0.1812	0.152*	
C33	0.0508 (5)	0.8748 (6)	0.3593 (3)	0.050 (2)	
C34	0.0084 (4)	0.8749 (5)	0.3994 (3)	0.049 (2)	
C35	0.0254 (5)	0.8130 (5)	0.4410 (3)	0.052 (2)	
H35	0.0608	0.7679	0.4421	0.063*	
C36	−0.0095 (5)	0.8176 (6)	0.4805 (3)	0.056 (2)	
H36	0.0037	0.7764	0.5086	0.067*	
C37	−0.0637 (5)	0.8822 (6)	0.4793 (3)	0.053 (2)	
C38	−0.0816 (5)	0.9422 (6)	0.4381 (3)	0.061 (2)	
H38	−0.1182	0.9861	0.4366	0.073*	
C39	−0.0458 (5)	0.9385 (6)	0.3985 (3)	0.058 (2)	
H39	−0.0589	0.9803	0.3707	0.070*	
C40	−0.1497 (5)	0.9489 (6)	0.5211 (4)	0.069 (3)	
H40A	−0.1252	1.0055	0.5228	0.103*	
H40B	−0.1659	0.9413	0.5526	0.103*	
H40C	−0.1946	0.9454	0.4887	0.103*	
C41	0.5420 (4)	0.8565 (5)	0.3748 (3)	0.0450 (19)	
C42	0.6159 (4)	0.8032 (5)	0.3929 (3)	0.0469 (19)	
C43	0.6462 (5)	0.7679 (6)	0.3556 (3)	0.058 (2)	
H43	0.6193	0.7747	0.3185	0.070*	
C44	0.7166 (5)	0.7218 (6)	0.3724 (4)	0.063 (2)	
H44	0.7367	0.6982	0.3466	0.076*	
C45	0.7564 (5)	0.7113 (6)	0.4265 (4)	0.062 (2)	
C46	0.7268 (5)	0.7464 (6)	0.4639 (4)	0.063 (2)	
H46	0.7540	0.7396	0.5009	0.076*	
C47	0.6571 (5)	0.7917 (6)	0.4477 (3)	0.058 (2)	
H47	0.6374	0.8148	0.4738	0.069*	
C48	0.8652 (6)	0.6417 (7)	0.4097 (4)	0.090 (3)	
H48A	0.8345	0.5972	0.3858	0.136*	
H48B	0.9160	0.6182	0.4293	0.136*	
H48C	0.8708	0.6919	0.3887	0.136*	
C49	0.0430 (5)	1.0567 (6)	0.2093 (3)	0.053 (2)	
H49	0.0710	1.0936	0.2376	0.064*	
C50	−0.0062 (5)	1.0956 (6)	0.1621 (3)	0.061 (2)	
H50	−0.0118	1.1567	0.1591	0.073*	
C51	−0.0459 (5)	1.0419 (6)	0.1204 (3)	0.062 (2)	
H51	−0.0782	1.0664	0.0880	0.075*	
C52	−0.0387 (5)	0.9507 (6)	0.1256 (3)	0.054 (2)	
C53	0.0119 (4)	0.9164 (5)	0.1751 (3)	0.0460 (19)	
C54	0.0210 (4)	0.8236 (5)	0.1827 (3)	0.0458 (19)	
C55	−0.0195 (4)	0.7657 (6)	0.1403 (3)	0.051 (2)	
C56	−0.0087 (5)	0.6753 (6)	0.1504 (3)	0.056 (2)	
H56	−0.0348	0.6355	0.1233	0.067*	
C57	0.0387 (5)	0.6447 (6)	0.1986 (3)	0.056 (2)	
H57	0.0455	0.5844	0.2052	0.067*	
C58	0.0773 (4)	0.7063 (5)	0.2385 (3)	0.051 (2)	
H58	0.1096	0.6854	0.2720	0.061*	
C59	−0.0784 (5)	0.8895 (6)	0.0834 (3)	0.062 (2)	
H59	−0.1108	0.9113	0.0502	0.074*	
C60	−0.0703 (5)	0.8030 (6)	0.0904 (3)	0.060 (2)	
H60	−0.0981	0.7656	0.0623	0.072*	
C61	0.4916 (5)	1.0356 (6)	0.2531 (3)	0.053 (2)	
H61	0.4789	0.9787	0.2396	0.063*	
C62	0.5217 (5)	1.0929 (6)	0.2238 (3)	0.056 (2)	
H62	0.5301	1.0743	0.1920	0.067*	
C63	0.5389 (5)	1.1761 (6)	0.2419 (3)	0.056 (2)	
H63	0.5568	1.2165	0.2217	0.067*	
C64	0.5297 (5)	1.2019 (5)	0.2917 (3)	0.052 (2)	
C65	0.5005 (4)	1.1385 (5)	0.3195 (3)	0.0455 (19)	
C66	0.4962 (4)	1.1582 (5)	0.3725 (3)	0.0449 (18)	
C67	0.5231 (5)	1.2403 (5)	0.3963 (3)	0.050 (2)	
C68	0.5279 (5)	1.2523 (6)	0.4510 (3)	0.056 (2)	
H68	0.5469	1.3052	0.4686	0.067*	
C69	0.5050 (5)	1.1869 (5)	0.4780 (3)	0.053 (2)	
H69	0.5093	1.1936	0.5145	0.063*	
C70	0.4751 (4)	1.1101 (5)	0.4502 (3)	0.0453 (18)	
H70	0.4572	1.0667	0.4686	0.054*	
C71	0.5517 (5)	1.2869 (6)	0.3160 (4)	0.060 (2)	
H71	0.5689	1.3300	0.2971	0.072*	
C72	0.5479 (5)	1.3052 (6)	0.3655 (4)	0.061 (2)	
H72	0.5617	1.3612	0.3802	0.074*	

### Atomic displacement parameters (Å^2^)

**Table:**

	*U*^11^	*U*^22^	*U*^33^	*U*^12^	*U*^13^	*U*^23^
Tb1	0.03549 (19)	0.02730 (19)	0.03401 (19)	−0.00289 (15)	0.00775 (14)	−0.00087 (15)
Tb2	0.03429 (19)	0.0311 (2)	0.03439 (19)	−0.00118 (15)	0.00943 (14)	−0.00004 (15)
N1	0.045 (4)	0.045 (4)	0.046 (4)	0.003 (3)	0.013 (3)	−0.001 (3)
N2	0.043 (4)	0.043 (4)	0.048 (4)	−0.004 (3)	0.015 (3)	−0.005 (3)
N3	0.048 (4)	0.044 (4)	0.044 (4)	−0.003 (3)	0.019 (3)	0.001 (3)
N4	0.045 (4)	0.037 (4)	0.045 (4)	−0.003 (3)	0.018 (3)	0.000 (3)
O1	0.048 (3)	0.033 (3)	0.056 (3)	0.001 (2)	0.007 (3)	−0.002 (2)
O2	0.052 (3)	0.039 (3)	0.053 (3)	−0.002 (3)	0.015 (3)	0.001 (2)
O3	0.095 (5)	0.054 (5)	0.101 (5)	0.016 (4)	0.015 (4)	0.023 (4)
O4	0.052 (3)	0.045 (3)	0.044 (3)	−0.003 (3)	0.009 (3)	0.002 (2)
O5	0.047 (3)	0.043 (3)	0.044 (3)	−0.008 (2)	0.020 (2)	−0.001 (2)
O6	0.086 (4)	0.056 (4)	0.051 (3)	−0.004 (3)	0.030 (3)	0.004 (3)
O7	0.050 (3)	0.051 (4)	0.044 (3)	0.002 (3)	0.018 (3)	0.000 (2)
O8	0.048 (3)	0.053 (4)	0.042 (3)	0.002 (3)	0.012 (3)	0.000 (2)
O9	0.080 (5)	0.153 (7)	0.048 (4)	0.024 (5)	0.015 (4)	0.005 (4)
O10	0.051 (3)	0.039 (3)	0.051 (3)	0.006 (3)	0.017 (3)	0.004 (2)
O11	0.050 (3)	0.040 (3)	0.053 (3)	0.006 (3)	0.013 (3)	0.001 (2)
O12	0.089 (5)	0.050 (4)	0.099 (5)	0.000 (4)	0.027 (4)	0.020 (4)
O13	0.055 (3)	0.056 (4)	0.059 (3)	0.008 (3)	0.025 (3)	0.013 (3)
O14	0.054 (3)	0.044 (3)	0.056 (3)	−0.001 (3)	0.021 (3)	0.002 (3)
O15	0.062 (4)	0.067 (4)	0.062 (4)	0.025 (3)	0.031 (3)	0.022 (3)
O16	0.046 (3)	0.048 (3)	0.046 (3)	0.005 (3)	0.012 (2)	−0.004 (3)
O17	0.047 (3)	0.053 (4)	0.046 (3)	0.007 (3)	0.014 (3)	0.002 (3)
O18	0.056 (4)	0.082 (5)	0.084 (5)	0.024 (3)	0.012 (3)	0.004 (4)
C1	0.048 (5)	0.036 (5)	0.048 (5)	−0.002 (4)	0.016 (4)	0.000 (4)
C2	0.051 (5)	0.032 (4)	0.054 (5)	0.002 (4)	0.020 (4)	0.004 (4)
C3	0.062 (6)	0.041 (5)	0.063 (5)	0.004 (4)	0.018 (5)	0.003 (4)
C4	0.070 (6)	0.050 (6)	0.072 (6)	0.012 (5)	0.012 (5)	0.010 (5)
C5	0.078 (7)	0.042 (6)	0.076 (7)	0.012 (5)	0.023 (5)	0.013 (5)
C6	0.075 (7)	0.042 (6)	0.077 (6)	0.001 (5)	0.016 (5)	0.011 (5)
C7	0.065 (6)	0.039 (5)	0.067 (6)	0.003 (4)	0.012 (4)	0.005 (4)
C8	0.116 (10)	0.062 (8)	0.135 (11)	0.013 (7)	0.024 (8)	0.036 (7)
C9	0.047 (5)	0.035 (4)	0.042 (4)	0.000 (3)	0.015 (4)	0.000 (3)
C10	0.042 (4)	0.037 (4)	0.039 (4)	0.001 (3)	0.014 (3)	0.001 (3)
C11	0.048 (5)	0.040 (5)	0.042 (4)	−0.001 (4)	0.014 (4)	0.000 (4)
C12	0.056 (5)	0.045 (5)	0.044 (5)	0.001 (4)	0.014 (4)	−0.001 (4)
C13	0.061 (5)	0.047 (5)	0.042 (4)	0.006 (4)	0.025 (4)	0.002 (4)
C14	0.061 (5)	0.049 (5)	0.048 (5)	−0.007 (4)	0.024 (4)	0.004 (4)
C15	0.053 (5)	0.045 (5)	0.045 (5)	−0.004 (4)	0.015 (4)	0.003 (4)
C16	0.122 (9)	0.068 (7)	0.069 (6)	−0.004 (6)	0.046 (6)	0.007 (5)
C17	0.048 (5)	0.052 (5)	0.043 (5)	0.005 (4)	0.018 (4)	0.000 (4)
C18	0.046 (5)	0.065 (6)	0.038 (4)	0.013 (4)	0.016 (4)	0.006 (4)
C19	0.055 (5)	0.087 (7)	0.045 (5)	0.009 (5)	0.017 (4)	0.012 (5)
C20	0.060 (6)	0.107 (9)	0.053 (6)	0.014 (6)	0.018 (5)	0.012 (5)
C21	0.066 (7)	0.114 (9)	0.043 (5)	0.021 (6)	0.012 (5)	0.009 (5)
C22	0.056 (6)	0.100 (8)	0.046 (5)	0.016 (5)	0.015 (4)	0.004 (5)
C23	0.051 (5)	0.079 (7)	0.043 (5)	0.009 (5)	0.016 (4)	0.004 (4)
C24	0.105 (10)	0.198 (16)	0.066 (7)	0.021 (10)	0.031 (7)	0.008 (8)
C25	0.048 (5)	0.037 (5)	0.048 (4)	0.002 (4)	0.021 (4)	0.000 (4)
C26	0.049 (5)	0.032 (4)	0.058 (5)	0.001 (4)	0.023 (4)	0.004 (4)
C27	0.058 (5)	0.041 (5)	0.072 (6)	0.001 (4)	0.028 (4)	0.009 (4)
C28	0.068 (6)	0.047 (6)	0.083 (6)	−0.003 (5)	0.035 (5)	0.013 (5)
C29	0.071 (6)	0.040 (5)	0.077 (6)	0.006 (5)	0.024 (5)	0.014 (5)
C30	0.062 (6)	0.043 (6)	0.083 (7)	0.006 (4)	0.025 (5)	0.009 (5)
C31	0.055 (5)	0.037 (5)	0.073 (6)	0.001 (4)	0.025 (4)	0.006 (4)
C32	0.120 (10)	0.073 (8)	0.118 (10)	0.004 (7)	0.048 (8)	0.027 (7)
C33	0.048 (5)	0.049 (6)	0.054 (5)	0.000 (4)	0.018 (4)	0.011 (4)
C34	0.048 (5)	0.050 (5)	0.053 (5)	0.006 (4)	0.021 (4)	0.012 (4)
C35	0.053 (5)	0.050 (5)	0.059 (5)	0.011 (4)	0.025 (4)	0.013 (4)
C36	0.058 (5)	0.058 (6)	0.058 (5)	0.017 (4)	0.027 (4)	0.019 (4)
C37	0.053 (5)	0.055 (6)	0.057 (5)	0.015 (4)	0.027 (4)	0.018 (4)
C38	0.056 (5)	0.062 (6)	0.065 (6)	0.023 (4)	0.021 (5)	0.021 (5)
C39	0.058 (5)	0.058 (6)	0.061 (5)	0.014 (4)	0.024 (4)	0.019 (4)
C40	0.068 (6)	0.078 (7)	0.068 (6)	0.026 (5)	0.034 (5)	0.018 (5)
C41	0.042 (5)	0.046 (5)	0.049 (5)	0.000 (4)	0.017 (4)	0.004 (4)
C42	0.039 (4)	0.051 (5)	0.052 (5)	0.007 (4)	0.017 (4)	0.002 (4)
C43	0.049 (5)	0.064 (6)	0.058 (5)	0.012 (4)	0.013 (4)	0.001 (4)
C44	0.053 (5)	0.069 (7)	0.066 (6)	0.017 (5)	0.019 (5)	−0.003 (5)
C45	0.048 (5)	0.064 (6)	0.069 (6)	0.014 (4)	0.013 (5)	0.007 (5)
C46	0.052 (5)	0.069 (6)	0.063 (6)	0.016 (5)	0.011 (4)	0.007 (5)
C47	0.048 (5)	0.065 (6)	0.058 (5)	0.012 (4)	0.015 (4)	0.003 (4)
C48	0.065 (7)	0.091 (9)	0.106 (9)	0.023 (6)	0.016 (6)	−0.007 (7)
C49	0.055 (5)	0.046 (5)	0.052 (5)	0.005 (4)	0.010 (4)	0.000 (4)
C50	0.063 (6)	0.056 (6)	0.054 (5)	0.010 (5)	0.005 (4)	0.003 (5)
C51	0.062 (6)	0.063 (6)	0.051 (5)	0.007 (5)	0.003 (4)	0.001 (5)
C52	0.053 (5)	0.057 (6)	0.045 (5)	0.003 (4)	0.009 (4)	−0.008 (4)
C53	0.045 (5)	0.052 (5)	0.039 (4)	0.000 (4)	0.012 (4)	−0.004 (4)
C54	0.044 (5)	0.052 (5)	0.041 (4)	−0.002 (4)	0.015 (4)	−0.008 (4)
C55	0.048 (5)	0.054 (6)	0.047 (5)	−0.002 (4)	0.013 (4)	−0.010 (4)
C56	0.053 (5)	0.055 (6)	0.054 (5)	−0.005 (4)	0.009 (4)	−0.018 (4)
C57	0.052 (5)	0.049 (5)	0.061 (6)	−0.009 (4)	0.013 (4)	−0.013 (4)
C58	0.049 (5)	0.044 (5)	0.055 (5)	−0.006 (4)	0.011 (4)	−0.006 (4)
C59	0.060 (6)	0.066 (7)	0.049 (5)	0.003 (5)	0.003 (4)	−0.004 (5)
C60	0.056 (5)	0.063 (7)	0.051 (5)	−0.003 (5)	0.005 (4)	−0.014 (5)
C61	0.057 (5)	0.053 (6)	0.050 (5)	−0.006 (4)	0.022 (4)	−0.002 (4)
C62	0.062 (6)	0.059 (6)	0.052 (5)	−0.005 (4)	0.029 (4)	0.005 (4)
C63	0.061 (5)	0.055 (6)	0.056 (5)	−0.006 (4)	0.025 (4)	0.012 (4)
C64	0.057 (5)	0.048 (5)	0.054 (5)	−0.005 (4)	0.021 (4)	0.007 (4)
C65	0.048 (5)	0.040 (5)	0.050 (5)	−0.002 (4)	0.019 (4)	0.005 (4)
C66	0.048 (5)	0.039 (5)	0.050 (5)	−0.003 (4)	0.019 (4)	0.004 (4)
C67	0.056 (5)	0.040 (5)	0.055 (5)	−0.005 (4)	0.020 (4)	0.000 (4)
C68	0.064 (6)	0.042 (5)	0.059 (5)	−0.007 (4)	0.018 (4)	−0.009 (4)
C69	0.060 (5)	0.046 (5)	0.052 (5)	−0.005 (4)	0.019 (4)	−0.006 (4)
C70	0.049 (5)	0.040 (5)	0.048 (5)	−0.004 (4)	0.017 (4)	−0.001 (4)
C71	0.070 (6)	0.049 (6)	0.063 (6)	−0.007 (4)	0.022 (5)	0.008 (4)
C72	0.071 (6)	0.047 (6)	0.065 (6)	−0.009 (4)	0.020 (5)	0.000 (4)

### Geometric parameters (Å, °)

**Table:**

Tb1—O4	2.255 (5)	C23—H23	0.9300
Tb1—O7	2.281 (5)	C24—H24A	0.9600
Tb1—O10	2.357 (5)	C24—H24B	0.9600
Tb1—O1	2.367 (5)	C24—H24C	0.9600
Tb1—O13	2.373 (5)	C25—C26	1.481 (10)
Tb1—O14	2.469 (5)	C26—C27	1.377 (10)
Tb1—N2	2.575 (6)	C26—C31	1.398 (10)
Tb1—N1	2.624 (6)	C27—C28	1.394 (11)
Tb2—O11	2.286 (5)	C27—H27	0.9300
Tb2—O2	2.300 (5)	C28—C29	1.365 (11)
Tb2—O5	2.357 (4)	C28—H28	0.9300
Tb2—O8	2.381 (5)	C29—C30	1.363 (11)
Tb2—O16	2.405 (5)	C30—C31	1.364 (11)
Tb2—O17	2.460 (5)	C30—H30	0.9300
Tb2—N3	2.582 (6)	C31—H31	0.9300
Tb2—N4	2.610 (6)	C32—H32A	0.9600
N1—C49	1.329 (9)	C32—H32B	0.9600
N1—C53	1.360 (9)	C32—H32C	0.9600
N2—C58	1.327 (9)	C33—C34	1.479 (10)
N2—C54	1.360 (9)	C34—C39	1.369 (10)
N3—C61	1.336 (9)	C34—C35	1.387 (10)
N3—C65	1.348 (9)	C35—C36	1.368 (10)
N4—C70	1.317 (9)	C35—H35	0.9300
N4—C66	1.360 (9)	C36—C37	1.377 (10)
O1—C1	1.260 (8)	C36—H36	0.9300
O2—C1	1.254 (8)	C37—C38	1.359 (10)
O3—C5	1.378 (10)	C38—C39	1.382 (11)
O3—C8	1.430 (12)	C38—H38	0.9300
O4—C9	1.267 (8)	C39—H39	0.9300
O5—C9	1.255 (8)	C40—H40A	0.9600
O6—C13	1.381 (8)	C40—H40B	0.9600
O6—C16	1.408 (10)	C40—H40C	0.9600
O7—C17	1.250 (8)	C41—C42	1.497 (10)
O8—C17	1.255 (9)	C42—C43	1.368 (10)
O9—C21	1.378 (10)	C42—C47	1.383 (10)
O9—C24	1.412 (12)	C43—C44	1.390 (10)
O10—C25	1.277 (8)	C43—H43	0.9300
O11—C25	1.252 (8)	C44—C45	1.363 (11)
O12—C29	1.366 (10)	C44—H44	0.9300
O12—C32	1.415 (11)	C45—C46	1.360 (11)
O13—C33	1.273 (9)	C46—C47	1.372 (10)
O14—C33	1.260 (9)	C46—H46	0.9300
O15—C37	1.353 (9)	C47—H47	0.9300
O15—C40	1.430 (9)	C48—H48A	0.9600
O16—C41	1.264 (8)	C48—H48B	0.9600
O17—C41	1.261 (8)	C48—H48C	0.9600
O18—C45	1.376 (9)	C49—C50	1.390 (10)
O18—C48	1.427 (11)	C49—H49	0.9300
C1—C2	1.485 (10)	C50—C51	1.356 (11)
C2—C7	1.380 (11)	C50—H50	0.9300
C2—C3	1.381 (10)	C51—C52	1.390 (11)
C3—C4	1.374 (11)	C51—H51	0.9300
C3—H3	0.9300	C52—C53	1.410 (10)
C4—C5	1.390 (12)	C52—C59	1.434 (11)
C4—H4	0.9300	C53—C54	1.422 (11)
C5—C6	1.342 (12)	C54—C55	1.410 (10)
C6—C7	1.382 (11)	C55—C56	1.394 (11)
C6—H6	0.9300	C55—C60	1.437 (11)
C7—H7	0.9300	C56—C57	1.347 (11)
C8—H8A	0.9600	C56—H56	0.9300
C8—H8B	0.9600	C57—C58	1.399 (10)
C8—H8C	0.9600	C57—H57	0.9300
C9—C10	1.489 (9)	C58—H58	0.9300
C10—C15	1.377 (10)	C59—C60	1.324 (11)
C10—C11	1.384 (10)	C59—H59	0.9300
C11—C12	1.370 (9)	C60—H60	0.9300
C11—H11	0.9300	C61—C62	1.378 (10)
C12—C13	1.366 (10)	C61—H61	0.9300
C12—H12	0.9300	C62—C63	1.346 (11)
C13—C14	1.379 (10)	C62—H62	0.9300
C14—C15	1.393 (10)	C63—C64	1.410 (11)
C14—H14	0.9300	C63—H63	0.9300
C15—H15	0.9300	C64—C65	1.401 (10)
C16—H16A	0.9600	C64—C71	1.431 (11)
C16—H16B	0.9600	C65—C66	1.435 (10)
C16—H16C	0.9600	C66—C67	1.402 (10)
C17—C18	1.494 (10)	C67—C68	1.406 (10)
C18—C19	1.372 (10)	C67—C72	1.428 (11)
C18—C23	1.385 (11)	C68—C69	1.354 (11)
C19—C20	1.391 (11)	C68—H68	0.9300
C19—H19	0.9300	C69—C70	1.383 (10)
C20—C21	1.381 (13)	C69—H69	0.9300
C20—H20	0.9300	C70—H70	0.9300
C21—C22	1.361 (12)	C71—C72	1.338 (11)
C22—C23	1.368 (10)	C71—H71	0.9300
C22—H22	0.9300	C72—H72	0.9300
			
O4—Tb1—O7	109.32 (18)	C18—C23—H23	118.7
O4—Tb1—O10	77.66 (17)	O9—C24—H24A	109.5
O7—Tb1—O10	79.12 (17)	O9—C24—H24B	109.5
O4—Tb1—O1	76.26 (17)	H24A—C24—H24B	109.5
O7—Tb1—O1	77.51 (18)	O9—C24—H24C	109.5
O10—Tb1—O1	136.51 (17)	H24A—C24—H24C	109.5
O4—Tb1—O13	92.76 (18)	H24B—C24—H24C	109.5
O7—Tb1—O13	146.47 (18)	O11—C25—O10	123.8 (7)
O10—Tb1—O13	81.51 (18)	O11—C25—C26	118.2 (7)
O1—Tb1—O13	133.68 (18)	O10—C25—C26	117.9 (7)
O4—Tb1—O14	78.98 (17)	C27—C26—C31	117.4 (7)
O7—Tb1—O14	153.05 (18)	C27—C26—C25	120.7 (7)
O10—Tb1—O14	127.78 (17)	C31—C26—C25	121.9 (7)
O1—Tb1—O14	79.88 (18)	C26—C27—C28	120.9 (8)
O13—Tb1—O14	53.80 (18)	C26—C27—H27	119.5
O4—Tb1—N2	144.37 (19)	C28—C27—H27	119.5
O7—Tb1—N2	83.94 (18)	C29—C28—C27	120.0 (8)
O10—Tb1—N2	137.97 (19)	C29—C28—H28	120.0
O1—Tb1—N2	74.66 (18)	C27—C28—H28	120.0
O13—Tb1—N2	92.62 (19)	C30—C29—C28	119.8 (8)
O14—Tb1—N2	76.05 (18)	C30—C29—O12	115.4 (8)
O4—Tb1—N1	151.71 (19)	C28—C29—O12	124.8 (8)
O7—Tb1—N1	74.56 (18)	C29—C30—C31	120.6 (8)
O10—Tb1—N1	75.64 (18)	C29—C30—H30	119.7
O1—Tb1—N1	130.75 (18)	C31—C30—H30	119.7
O13—Tb1—N1	74.27 (18)	C30—C31—C26	121.2 (8)
O14—Tb1—N1	110.68 (18)	C30—C31—H31	119.4
N2—Tb1—N1	62.8 (2)	C26—C31—H31	119.4
O11—Tb2—O2	114.01 (18)	O12—C32—H32A	109.5
O11—Tb2—O5	81.14 (17)	O12—C32—H32B	109.5
O2—Tb2—O5	78.68 (17)	H32A—C32—H32B	109.5
O11—Tb2—O8	76.29 (17)	O12—C32—H32C	109.5
O2—Tb2—O8	80.42 (17)	H32A—C32—H32C	109.5
O5—Tb2—O8	139.48 (17)	H32B—C32—H32C	109.5
O11—Tb2—O16	149.78 (17)	O14—C33—O13	119.9 (7)
O2—Tb2—O16	83.65 (17)	O14—C33—C34	121.2 (7)
O5—Tb2—O16	78.59 (17)	O13—C33—C34	118.8 (7)
O8—Tb2—O16	132.73 (17)	C39—C34—C35	117.5 (7)
O11—Tb2—O17	150.30 (17)	C39—C34—C33	121.4 (7)
O2—Tb2—O17	78.26 (17)	C35—C34—C33	121.0 (7)
O5—Tb2—O17	128.53 (17)	C36—C35—C34	120.8 (7)
O8—Tb2—O17	79.59 (17)	C36—C35—H35	119.6
O16—Tb2—O17	53.60 (16)	C34—C35—H35	119.6
O11—Tb2—N3	84.77 (19)	C35—C36—C37	121.1 (7)
O2—Tb2—N3	142.89 (18)	C35—C36—H36	119.4
O5—Tb2—N3	137.67 (18)	C37—C36—H36	119.4
O8—Tb2—N3	73.18 (18)	O15—C37—C38	124.9 (7)
O16—Tb2—N3	95.37 (18)	O15—C37—C36	116.7 (7)
O17—Tb2—N3	71.76 (18)	C38—C37—C36	118.4 (7)
O11—Tb2—N4	75.08 (18)	C37—C38—C39	120.7 (8)
O2—Tb2—N4	150.43 (18)	C37—C38—H38	119.7
O5—Tb2—N4	75.02 (17)	C39—C38—H38	119.7
O8—Tb2—N4	128.85 (18)	C34—C39—C38	121.5 (8)
O16—Tb2—N4	78.16 (18)	C34—C39—H39	119.3
O17—Tb2—N4	108.14 (18)	C38—C39—H39	119.3
N3—Tb2—N4	62.80 (18)	O15—C40—H40A	109.5
C49—N1—C53	117.6 (7)	O15—C40—H40B	109.5
C49—N1—Tb1	123.0 (5)	H40A—C40—H40B	109.5
C53—N1—Tb1	119.2 (5)	O15—C40—H40C	109.5
C58—N2—C54	118.2 (7)	H40A—C40—H40C	109.5
C58—N2—Tb1	120.8 (5)	H40B—C40—H40C	109.5
C54—N2—Tb1	120.8 (5)	O17—C41—O16	120.6 (7)
C61—N3—C65	117.0 (6)	O17—C41—C42	120.3 (7)
C61—N3—Tb2	122.4 (5)	O16—C41—C42	119.0 (7)
C65—N3—Tb2	120.1 (5)	C43—C42—C47	118.2 (7)
C70—N4—C66	117.8 (6)	C43—C42—C41	120.6 (7)
C70—N4—Tb2	123.9 (5)	C47—C42—C41	121.1 (7)
C66—N4—Tb2	118.1 (5)	C42—C43—C44	120.7 (8)
C1—O1—Tb1	130.2 (5)	C42—C43—H43	119.6
C1—O2—Tb2	156.9 (5)	C44—C43—H43	119.6
C5—O3—C8	116.8 (8)	C45—C44—C43	120.2 (8)
C9—O4—Tb1	163.0 (5)	C45—C44—H44	119.9
C9—O5—Tb2	127.3 (4)	C43—C44—H44	119.9
C13—O6—C16	117.5 (7)	C46—C45—C44	119.4 (8)
C17—O7—Tb1	161.1 (5)	C46—C45—O18	115.7 (8)
C17—O8—Tb2	127.9 (5)	C44—C45—O18	124.8 (8)
C21—O9—C24	118.4 (8)	C45—C46—C47	120.8 (8)
C25—O10—Tb1	130.0 (5)	C45—C46—H46	119.6
C25—O11—Tb2	157.2 (5)	C47—C46—H46	119.6
C29—O12—C32	117.8 (8)	C46—C47—C42	120.6 (8)
C33—O13—Tb1	94.5 (5)	C46—C47—H47	119.7
C33—O14—Tb1	90.4 (5)	C42—C47—H47	119.7
C37—O15—C40	118.2 (6)	O18—C48—H48A	109.5
C41—O16—Tb2	94.1 (4)	O18—C48—H48B	109.5
C41—O17—Tb2	91.6 (4)	H48A—C48—H48B	109.5
C45—O18—C48	117.1 (7)	O18—C48—H48C	109.5
O2—C1—O1	124.0 (7)	H48A—C48—H48C	109.5
O2—C1—C2	117.7 (7)	H48B—C48—H48C	109.5
O1—C1—C2	118.3 (7)	N1—C49—C50	124.2 (8)
C7—C2—C3	117.6 (7)	N1—C49—H49	117.9
C7—C2—C1	120.9 (7)	C50—C49—H49	117.9
C3—C2—C1	121.4 (7)	C51—C50—C49	118.0 (9)
C4—C3—C2	120.9 (8)	C51—C50—H50	121.0
C4—C3—H3	119.5	C49—C50—H50	121.0
C2—C3—H3	119.5	C50—C51—C52	120.7 (8)
C3—C4—C5	119.9 (9)	C50—C51—H51	119.7
C3—C4—H4	120.1	C52—C51—H51	119.7
C5—C4—H4	120.1	C51—C52—C53	117.8 (7)
C6—C5—O3	124.6 (9)	C51—C52—C59	124.0 (8)
C6—C5—C4	119.9 (8)	C53—C52—C59	118.1 (8)
O3—C5—C4	115.5 (9)	N1—C53—C52	121.7 (7)
C5—C6—C7	120.1 (9)	N1—C53—C54	118.2 (7)
C5—C6—H6	120.0	C52—C53—C54	120.1 (7)
C7—C6—H6	120.0	N2—C54—C55	121.7 (7)
C2—C7—C6	121.5 (8)	N2—C54—C53	118.3 (7)
C2—C7—H7	119.2	C55—C54—C53	120.0 (7)
C6—C7—H7	119.2	C56—C55—C54	117.3 (7)
O3—C8—H8A	109.5	C56—C55—C60	124.3 (8)
O3—C8—H8B	109.5	C54—C55—C60	118.4 (8)
H8A—C8—H8B	109.5	C57—C56—C55	121.3 (8)
O3—C8—H8C	109.5	C57—C56—H56	119.3
H8A—C8—H8C	109.5	C55—C56—H56	119.3
H8B—C8—H8C	109.5	C56—C57—C58	118.0 (8)
O5—C9—O4	123.8 (7)	C56—C57—H57	121.0
O5—C9—C10	119.4 (7)	C58—C57—H57	121.0
O4—C9—C10	116.8 (7)	N2—C58—C57	123.5 (8)
C15—C10—C11	118.4 (7)	N2—C58—H58	118.2
C15—C10—C9	119.8 (7)	C57—C58—H58	118.2
C11—C10—C9	121.8 (7)	C60—C59—C52	122.0 (8)
C12—C11—C10	121.0 (7)	C60—C59—H59	119.0
C12—C11—H11	119.5	C52—C59—H59	119.0
C10—C11—H11	119.5	C59—C60—C55	121.4 (8)
C13—C12—C11	119.9 (7)	C59—C60—H60	119.3
C13—C12—H12	120.1	C55—C60—H60	119.3
C11—C12—H12	120.1	N3—C61—C62	124.1 (8)
C12—C13—C14	120.9 (7)	N3—C61—H61	117.9
C12—C13—O6	114.7 (7)	C62—C61—H61	117.9
C14—C13—O6	124.4 (7)	C63—C62—C61	118.9 (8)
C13—C14—C15	118.5 (7)	C63—C62—H62	120.5
C13—C14—H14	120.8	C61—C62—H62	120.5
C15—C14—H14	120.8	C62—C63—C64	119.8 (7)
C10—C15—C14	121.2 (7)	C62—C63—H63	120.1
C10—C15—H15	119.4	C64—C63—H63	120.1
C14—C15—H15	119.4	C65—C64—C63	117.3 (8)
O6—C16—H16A	109.5	C65—C64—C71	119.0 (7)
O6—C16—H16B	109.5	C63—C64—C71	123.6 (7)
H16A—C16—H16B	109.5	N3—C65—C64	122.7 (7)
O6—C16—H16C	109.5	N3—C65—C66	117.3 (6)
H16A—C16—H16C	109.5	C64—C65—C66	119.9 (7)
H16B—C16—H16C	109.5	N4—C66—C67	122.1 (7)
O7—C17—O8	125.2 (7)	N4—C66—C65	118.7 (7)
O7—C17—C18	116.8 (7)	C67—C66—C65	119.2 (7)
O8—C17—C18	118.0 (7)	C66—C67—C68	117.3 (7)
C19—C18—C23	117.4 (7)	C66—C67—C72	119.3 (7)
C19—C18—C17	122.1 (7)	C68—C67—C72	123.3 (8)
C23—C18—C17	120.4 (7)	C69—C68—C67	120.0 (8)
C18—C19—C20	121.4 (8)	C69—C68—H68	120.0
C18—C19—H19	119.3	C67—C68—H68	120.0
C20—C19—H19	119.3	C68—C69—C70	118.6 (8)
C21—C20—C19	118.5 (9)	C68—C69—H69	120.7
C21—C20—H20	120.7	C70—C69—H69	120.7
C19—C20—H20	120.7	N4—C70—C69	124.1 (7)
C22—C21—O9	115.0 (9)	N4—C70—H70	117.9
C22—C21—C20	121.3 (9)	C69—C70—H70	117.9
O9—C21—C20	123.7 (9)	C72—C71—C64	121.1 (8)
C21—C22—C23	118.5 (9)	C72—C71—H71	119.5
C21—C22—H22	120.8	C64—C71—H71	119.5
C23—C22—H22	120.8	C71—C72—C67	121.2 (8)
C22—C23—C18	122.7 (8)	C71—C72—H72	119.4
C22—C23—H23	118.7	C67—C72—H72	119.4
			
O4—Tb1—N1—C49	12.7 (8)	O4—C9—C10—C11	−153.3 (7)
O7—Tb1—N1—C49	−89.5 (6)	C15—C10—C11—C12	−1.4 (11)
O10—Tb1—N1—C49	−7.1 (6)	C9—C10—C11—C12	179.0 (7)
O1—Tb1—N1—C49	−147.2 (5)	C10—C11—C12—C13	3.8 (12)
O13—Tb1—N1—C49	78.0 (6)	C11—C12—C13—C14	−3.6 (12)
O14—Tb1—N1—C49	118.3 (6)	C11—C12—C13—O6	177.5 (7)
N2—Tb1—N1—C49	179.4 (6)	C16—O6—C13—C12	−170.2 (8)
O4—Tb1—N1—C53	−173.6 (5)	C16—O6—C13—C14	11.0 (12)
O7—Tb1—N1—C53	84.2 (5)	C12—C13—C14—C15	1.1 (12)
O10—Tb1—N1—C53	166.6 (5)	O6—C13—C14—C15	179.9 (7)
O1—Tb1—N1—C53	26.5 (6)	C11—C10—C15—C14	−1.1 (11)
O13—Tb1—N1—C53	−108.3 (5)	C9—C10—C15—C14	178.5 (7)
O14—Tb1—N1—C53	−68.0 (5)	C13—C14—C15—C10	1.3 (12)
N2—Tb1—N1—C53	−6.9 (5)	Tb1—O7—C17—O8	−1(2)
O4—Tb1—N2—C58	−9.1 (7)	Tb1—O7—C17—C18	177.3 (12)
O7—Tb1—N2—C58	105.9 (6)	Tb2—O8—C17—O7	−10.4 (11)
O10—Tb1—N2—C58	172.2 (5)	Tb2—O8—C17—C18	171.7 (5)
O1—Tb1—N2—C58	27.2 (5)	O7—C17—C18—C19	169.3 (8)
O13—Tb1—N2—C58	−107.5 (6)	O8—C17—C18—C19	−12.7 (12)
O14—Tb1—N2—C58	−55.9 (5)	O7—C17—C18—C23	−13.5 (11)
N1—Tb1—N2—C58	−178.4 (6)	O8—C17—C18—C23	164.5 (8)
O4—Tb1—N2—C54	176.1 (5)	C23—C18—C19—C20	−2.5 (13)
O7—Tb1—N2—C54	−68.9 (5)	C17—C18—C19—C20	174.7 (8)
O10—Tb1—N2—C54	−2.6 (6)	C18—C19—C20—C21	−2.0 (15)
O1—Tb1—N2—C54	−147.6 (5)	C24—O9—C21—C22	−165.6 (10)
O13—Tb1—N2—C54	77.6 (5)	C24—O9—C21—C20	14.4 (16)
O14—Tb1—N2—C54	129.3 (5)	C19—C20—C21—C22	6.2 (16)
N1—Tb1—N2—C54	6.8 (5)	C19—C20—C21—O9	−173.9 (9)
O11—Tb2—N3—C61	−110.7 (6)	O9—C21—C22—C23	174.4 (8)
O2—Tb2—N3—C61	13.1 (7)	C20—C21—C22—C23	−5.7 (15)
O5—Tb2—N3—C61	178.6 (5)	C21—C22—C23—C18	0.9 (14)
O8—Tb2—N3—C61	−33.4 (6)	C19—C18—C23—C22	3.1 (13)
O16—Tb2—N3—C61	99.7 (6)	C17—C18—C23—C22	−174.2 (8)
O17—Tb2—N3—C61	50.9 (6)	Tb2—O11—C25—O10	−0.6 (18)
N4—Tb2—N3—C61	173.4 (6)	Tb2—O11—C25—C26	−177.5 (9)
O11—Tb2—N3—C65	61.2 (5)	Tb1—O10—C25—O11	−23.5 (10)
O2—Tb2—N3—C65	−175.0 (5)	Tb1—O10—C25—C26	153.3 (5)
O5—Tb2—N3—C65	−9.5 (7)	O11—C25—C26—C27	13.2 (11)
O8—Tb2—N3—C65	138.5 (6)	O10—C25—C26—C27	−163.8 (7)
O16—Tb2—N3—C65	−88.4 (5)	O11—C25—C26—C31	−168.6 (7)
O17—Tb2—N3—C65	−137.2 (6)	O10—C25—C26—C31	14.4 (11)
N4—Tb2—N3—C65	−14.7 (5)	C31—C26—C27—C28	−2.0 (12)
O11—Tb2—N4—C70	96.3 (6)	C25—C26—C27—C28	176.3 (8)
O2—Tb2—N4—C70	−16.2 (8)	C26—C27—C28—C29	0.9 (14)
O5—Tb2—N4—C70	11.7 (5)	C27—C28—C29—C30	0.5 (14)
O8—Tb2—N4—C70	154.4 (5)	C27—C28—C29—O12	−179.6 (8)
O16—Tb2—N4—C70	−69.5 (6)	C32—O12—C29—C30	−172.4 (9)
O17—Tb2—N4—C70	−114.5 (6)	C32—O12—C29—C28	7.7 (14)
N3—Tb2—N4—C70	−171.9 (6)	C28—C29—C30—C31	−0.7 (14)
O11—Tb2—N4—C66	−78.0 (5)	O12—C29—C30—C31	179.5 (8)
O2—Tb2—N4—C66	169.4 (5)	C29—C30—C31—C26	−0.5 (14)
O5—Tb2—N4—C66	−162.6 (5)	C27—C26—C31—C30	1.8 (12)
O8—Tb2—N4—C66	−20.0 (6)	C25—C26—C31—C30	−176.4 (8)
O16—Tb2—N4—C66	116.2 (5)	Tb1—O14—C33—O13	11.5 (8)
O17—Tb2—N4—C66	71.1 (5)	Tb1—O14—C33—C34	−164.5 (7)
N3—Tb2—N4—C66	13.7 (5)	Tb1—O13—C33—O14	−12.0 (8)
O4—Tb1—O1—C1	−39.0 (6)	Tb1—O13—C33—C34	164.1 (6)
O7—Tb1—O1—C1	74.8 (6)	O14—C33—C34—C39	−168.5 (8)
O10—Tb1—O1—C1	15.7 (7)	O13—C33—C34—C39	15.5 (12)
O13—Tb1—O1—C1	−119.5 (6)	O14—C33—C34—C35	14.9 (12)
O14—Tb1—O1—C1	−120.0 (6)	O13—C33—C34—C35	−161.1 (8)
N2—Tb1—O1—C1	161.8 (6)	C39—C34—C35—C36	−1.9 (13)
N1—Tb1—O1—C1	131.4 (6)	C33—C34—C35—C36	174.8 (8)
O11—Tb2—O2—C1	−2.2 (13)	C34—C35—C36—C37	1.7 (13)
O5—Tb2—O2—C1	72.7 (12)	C40—O15—C37—C38	−1.9 (13)
O8—Tb2—O2—C1	−72.4 (12)	C40—O15—C37—C36	177.9 (8)
O16—Tb2—O2—C1	152.3 (12)	C35—C36—C37—O15	179.6 (8)
O17—Tb2—O2—C1	−153.7 (13)	C35—C36—C37—C38	−0.6 (14)
N3—Tb2—O2—C1	−117.2 (12)	O15—C37—C38—C39	179.5 (8)
N4—Tb2—O2—C1	100.2 (12)	C36—C37—C38—C39	−0.3 (14)
O7—Tb1—O4—C9	18.8 (17)	C35—C34—C39—C38	1.1 (13)
O10—Tb1—O4—C9	−54.8 (17)	C33—C34—C39—C38	−175.6 (8)
O1—Tb1—O4—C9	90.1 (17)	C37—C38—C39—C34	0.0 (14)
O13—Tb1—O4—C9	−135.5 (17)	Tb2—O17—C41—O16	−1.8 (7)
O14—Tb1—O4—C9	172.2 (17)	Tb2—O17—C41—C42	179.1 (6)
N2—Tb1—O4—C9	126.1 (16)	Tb2—O16—C41—O17	1.8 (8)
N1—Tb1—O4—C9	−74.4 (18)	Tb2—O16—C41—C42	−179.0 (6)
O11—Tb2—O5—C9	70.3 (6)	O17—C41—C42—C43	3.1 (12)
O2—Tb2—O5—C9	−46.5 (6)	O16—C41—C42—C43	−176.1 (8)
O8—Tb2—O5—C9	13.8 (7)	O17—C41—C42—C47	−179.8 (7)
O16—Tb2—O5—C9	−132.2 (6)	O16—C41—C42—C47	1.0 (12)
O17—Tb2—O5—C9	−111.4 (6)	C47—C42—C43—C44	−0.2 (13)
N3—Tb2—O5—C9	142.4 (5)	C41—C42—C43—C44	177.0 (8)
N4—Tb2—O5—C9	147.2 (6)	C42—C43—C44—C45	0.1 (14)
O4—Tb1—O7—C17	4.2 (16)	C43—C44—C45—C46	−0.3 (14)
O10—Tb1—O7—C17	76.9 (16)	C43—C44—C45—O18	−179.1 (9)
O1—Tb1—O7—C17	−66.2 (16)	C48—O18—C45—C46	−171.1 (9)
O13—Tb1—O7—C17	132.7 (15)	C48—O18—C45—C44	7.7 (14)
O14—Tb1—O7—C17	−99.8 (16)	C44—C45—C46—C47	0.5 (15)
N2—Tb1—O7—C17	−141.7 (16)	O18—C45—C46—C47	179.4 (8)
N1—Tb1—O7—C17	154.8 (16)	C45—C46—C47—C42	−0.6 (14)
O11—Tb2—O8—C17	−51.0 (6)	C43—C42—C47—C46	0.4 (13)
O2—Tb2—O8—C17	66.7 (6)	C41—C42—C47—C46	−176.8 (8)
O5—Tb2—O8—C17	7.0 (7)	C53—N1—C49—C50	0.2 (12)
O16—Tb2—O8—C17	138.8 (6)	Tb1—N1—C49—C50	174.0 (6)
O17—Tb2—O8—C17	146.4 (6)	N1—C49—C50—C51	−1.3 (13)
N3—Tb2—O8—C17	−139.6 (6)	C49—C50—C51—C52	1.6 (13)
N4—Tb2—O8—C17	−108.6 (6)	C50—C51—C52—C53	−1.1 (13)
O4—Tb1—O10—C25	75.2 (6)	C50—C51—C52—C59	−179.8 (8)
O7—Tb1—O10—C25	−37.6 (6)	C49—N1—C53—C52	0.4 (11)
O1—Tb1—O10—C25	20.9 (7)	Tb1—N1—C53—C52	−173.7 (6)
O13—Tb1—O10—C25	169.9 (6)	C49—N1—C53—C54	−179.2 (7)
O14—Tb1—O10—C25	140.5 (6)	Tb1—N1—C53—C54	6.8 (9)
N2—Tb1—O10—C25	−105.7 (6)	C51—C52—C53—N1	0.0 (12)
N1—Tb1—O10—C25	−114.3 (6)	C59—C52—C53—N1	178.8 (7)
O2—Tb2—O11—C25	13.8 (13)	C51—C52—C53—C54	179.6 (7)
O5—Tb2—O11—C25	−59.6 (12)	C59—C52—C53—C54	−1.6 (11)
O8—Tb2—O11—C25	86.5 (13)	C58—N2—C54—C55	−2.7 (11)
O16—Tb2—O11—C25	−107.8 (12)	Tb1—N2—C54—C55	172.3 (5)
O17—Tb2—O11—C25	123.1 (12)	C58—N2—C54—C53	178.6 (7)
N3—Tb2—O11—C25	160.5 (13)	Tb1—N2—C54—C53	−6.5 (9)
N4—Tb2—O11—C25	−136.3 (13)	N1—C53—C54—N2	−0.3 (10)
O4—Tb1—O13—C33	−67.6 (5)	C52—C53—C54—N2	−179.9 (7)
O7—Tb1—O13—C33	160.1 (4)	N1—C53—C54—C55	−179.1 (7)
O10—Tb1—O13—C33	−144.7 (5)	C52—C53—C54—C55	1.3 (11)
O1—Tb1—O13—C33	5.9 (6)	N2—C54—C55—C56	1.8 (11)
O14—Tb1—O13—C33	6.6 (4)	C53—C54—C55—C56	−179.5 (7)
N2—Tb1—O13—C33	77.1 (5)	N2—C54—C55—C60	−179.8 (7)
N1—Tb1—O13—C33	137.9 (5)	C53—C54—C55—C60	−1.0 (11)
O4—Tb1—O14—C33	95.1 (5)	C54—C55—C56—C57	−0.3 (12)
O7—Tb1—O14—C33	−153.7 (5)	C60—C55—C56—C57	−178.6 (8)
O10—Tb1—O14—C33	30.4 (5)	C55—C56—C57—C58	−0.3 (12)
O1—Tb1—O14—C33	172.9 (5)	C54—N2—C58—C57	2.2 (11)
O13—Tb1—O14—C33	−6.6 (4)	Tb1—N2—C58—C57	−172.8 (6)
N2—Tb1—O14—C33	−110.5 (5)	C56—C57—C58—N2	−0.7 (12)
N1—Tb1—O14—C33	−57.2 (5)	C51—C52—C59—C60	−179.5 (9)
O11—Tb2—O16—C41	−152.4 (4)	C53—C52—C59—C60	1.8 (13)
O2—Tb2—O16—C41	79.1 (4)	C52—C59—C60—C55	−1.6 (14)
O5—Tb2—O16—C41	158.8 (5)	C56—C55—C60—C59	179.6 (8)
O8—Tb2—O16—C41	8.4 (5)	C54—C55—C60—C59	1.2 (13)
O17—Tb2—O16—C41	−1.0 (4)	C65—N3—C61—C62	−1.9 (12)
N3—Tb2—O16—C41	−63.6 (5)	Tb2—N3—C61—C62	170.2 (6)
N4—Tb2—O16—C41	−124.4 (5)	N3—C61—C62—C63	−1.5 (13)
O11—Tb2—O17—C41	151.9 (4)	C61—C62—C63—C64	3.2 (13)
O2—Tb2—O17—C41	−89.7 (4)	C62—C63—C64—C65	−1.6 (12)
O5—Tb2—O17—C41	−24.7 (5)	C62—C63—C64—C71	175.4 (8)
O8—Tb2—O17—C41	−172.0 (5)	C61—N3—C65—C64	3.6 (11)
O16—Tb2—O17—C41	1.0 (4)	Tb2—N3—C65—C64	−168.8 (6)
N3—Tb2—O17—C41	112.4 (5)	C61—N3—C65—C66	−173.0 (7)
N4—Tb2—O17—C41	60.4 (5)	Tb2—N3—C65—C66	14.6 (9)
Tb2—O2—C1—O1	13.9 (18)	C63—C64—C65—N3	−1.9 (12)
Tb2—O2—C1—C2	−165.7 (9)	C71—C64—C65—N3	−179.0 (7)
Tb1—O1—C1—O2	−25.2 (11)	C63—C64—C65—C66	174.6 (7)
Tb1—O1—C1—C2	154.4 (5)	C71—C64—C65—C66	−2.5 (12)
O2—C1—C2—C7	−167.2 (7)	C70—N4—C66—C67	−3.5 (11)
O1—C1—C2—C7	13.2 (11)	Tb2—N4—C66—C67	171.2 (6)
O2—C1—C2—C3	16.0 (11)	C70—N4—C66—C65	172.5 (7)
O1—C1—C2—C3	−163.6 (7)	Tb2—N4—C66—C65	−12.8 (9)
C7—C2—C3—C4	−1.7 (12)	N3—C65—C66—N4	−0.9 (11)
C1—C2—C3—C4	175.2 (7)	C64—C65—C66—N4	−177.6 (7)
C2—C3—C4—C5	0.5 (13)	N3—C65—C66—C67	175.2 (7)
C8—O3—C5—C6	−4.2 (14)	C64—C65—C66—C67	−1.5 (11)
C8—O3—C5—C4	174.3 (9)	N4—C66—C67—C68	4.6 (12)
C3—C4—C5—C6	2.0 (14)	C65—C66—C67—C68	−171.4 (7)
C3—C4—C5—O3	−176.6 (8)	N4—C66—C67—C72	−178.7 (7)
O3—C5—C6—C7	175.2 (9)	C65—C66—C67—C72	5.3 (12)
C4—C5—C6—C7	−3.2 (15)	C66—C67—C68—C69	−1.8 (12)
C3—C2—C7—C6	0.4 (13)	C72—C67—C68—C69	−178.4 (8)
C1—C2—C7—C6	−176.5 (8)	C67—C68—C69—C70	−1.7 (12)
C5—C6—C7—C2	2.1 (14)	C66—N4—C70—C69	−0.4 (11)
Tb2—O5—C9—O4	−13.6 (10)	Tb2—N4—C70—C69	−174.8 (6)
Tb2—O5—C9—C10	166.5 (5)	C68—C69—C70—N4	3.0 (12)
Tb1—O4—C9—O5	−10 (2)	C65—C64—C71—C72	2.8 (13)
Tb1—O4—C9—C10	169.5 (13)	C63—C64—C71—C72	−174.2 (8)
O5—C9—C10—C15	−153.0 (7)	C64—C71—C72—C67	1.1 (14)
O4—C9—C10—C15	27.2 (10)	C66—C67—C72—C71	−5.2 (13)
O5—C9—C10—C11	26.6 (11)	C68—C67—C72—C71	171.3 (8)

### Hydrogen-bond geometry (Å, °)

**Table:**

*D*—H···*A*	*D*—H	H···*A*	*D*···*A*	*D*—H···*A*
C32—H32B···O6^i^	0.96	2.51	3.059 (13)	116
C51—H51···O9^ii^	0.93	2.41	3.317 (10)	164
C56—H56···O15^iii^	0.93	2.56	3.341 (9)	143
C60—H60···O15^iii^	0.93	2.48	3.280 (10)	144
C69—H69···O16^iv^	0.93	2.52	3.285 (9)	139

Symmetry codes: (i) *x*, −*y*+5/2, *z*−1/2; (ii) −*x*, −*y*+2, −*z*; (iii) *x*, −*y*+3/2, *z*−1/2; (iv) −*x*+1, −*y*+2, −*z*+1.
